# The Endometrial Receptivity Test: The Impact of Combined Treatment with Pentoxifylline and Alpha-Tocopherol in Patients with Recurrent Implantation Failure or Recurrent Pregnancy Loss

**DOI:** 10.3390/jcm14165903

**Published:** 2025-08-21

**Authors:** Laurine Prudhomme, Cécile Habran, Soraya Labied, Frédéric Wenders, Laetitia Rousseau, Carine Munaut, Laurie Henry

**Affiliations:** 1 ART Center of the Department of Obstetrics and Gynecology, CHU of Liège-Citadelle Site, University of Liège, 4000 Liege, Belgium; cecile.habran@citadelle.be (C.H.); soraya.labied@citadelle.be (S.L.); frederic.wenders@citadelle.be (F.W.); laetitia.rousseau@citadelle.be (L.R.); laurie.henry@citadelle.be (L.H.); 2 Laboratory of Tumor and Development Biology, Giga-Cancer, University of Liège, 4000 Liege, Belgium; c.munaut@uliege.be

**Keywords:** implantation/miscarriages, tocopherol, pentoxifylline

## Abstract

**Background/Objectives**: The management of patients with recurrent implantation failure (RIF) or recurrent pregnancy loss (RPL) is a real challenge. Studying endometrial proliferation and vascularization by ultrasound during the embryo implantation window is an option for investigating these failures. This approach involves measuring the endometrial volume, the uterine arteries pulsatility index (PI), and the sub-endometrial flow index (VFI). **Methods**: The aim of our single-center retrospective study was to evaluate the benefit of treatment with pentoxifylline (400 mg twice daily) and alpha-tocopherol (500 IU twice daily), which was administered for at least 3 months. This study included 52 patients presenting abnormal ultrasound criteria, i.e., endometrial volume less than 2 cm^3^ and/or PI greater than 2.8 and/or VFI less than 0.25. **Results**: After treatment, we observed a significant increase in endometrial volume of 0.32 cm^3^ (*p* = 0.0054), as well as a significant increase in VFI of 0.49 (*p* = 0.041) in comparison to the control group. After treatment, the PI of the right uterine artery decreased significantly by 0.25 (*p* = 0.029) and the PI of the left uterine artery decreased by 0.27, but not significantly. In addition, our study showed that the clinical pregnancy rate (CPR) was more improved in the treated group compared to controls. **Conclusions**: Our study showed a promising benefit of pentoxifylline and alpha-tocopherol on endometrial properties; this needs to be corroborated by a larger prospective study.

## 1. Introduction

Embryo implantation failure is a major challenge in reproductive medicine and a leading cause of unsuccessful assisted reproductive technologies (ART). Despite advances in the field of in vitro fertilization (IVF) techniques, implantation remains a complex process involving synchronized embryo development, endometrial receptivity, and maternal-embryonic crosstalk [1]. Recurrent implantation failure (RIF), defined as the failure to achieve pregnancy after multiple embryo transfers, can result from various factors, including embryonic aneuploidy, uterine anomalies, immune dysfunction, and impaired endometrial receptivity or endometrial dysbiosis [2,3]. Among these, endometrial receptivity plays a critical role in ensuring successful implantation, and its assessment has become an area of growing interest in reproductive medicine.

The endometrial receptivity to the embryo during the implantation window can be assessed using various methods, including ultrasound (e.g., endometrial thickness, volume, vascularization, wave-like activity…), endometrial biopsy (e.g., histology markers, receptivity array), endometrial fluid aspiration, and hysteroscopy [4]. Such assessments are particularly relevant for women experiencing RIF during IVF cycles, but they can also be considered in patients with recurrent pregnancy loss (RPL) and, to a lesser extent, in patients with persistently thin endometrium during ART.

Among these methods, ultrasound evaluation of endometrial proliferation and vascularization has been proposed as a non-invasive tool for assessing endometrial receptivity. Previous studies have demonstrated the prognostic value of specific ultrasound parameters. In 2003, Zollner et al. reported that an endometrial volume of less than 2.5 cm^3^ was associated with significantly lower pregnancy rates after fresh embryo transfer compared to volumes greater than or equal to 2.5 cm^3^ [5]. Endometrial vascularity, assessed by the pulsatility index (PI) of the uterine arteries, also plays a key role in implantation, with a PI greater than 2.8 being linked to reduced pregnancy chances [6]. Additionally, the vascularization flow index (VFI), which reflects sub-endometrial tissue perfusion within 5 mm of the endometrial–myometrial junction, has been correlated with IVF success, with pregnancy rates (PRs) increasing when the VFI exceeds 0.24 [7,8].

Given the importance of endometrial vascularization in implantation, various therapeutic approaches have been explored to enhance endometrial receptivity.

Alpha-tocopherol is a well-known antioxidant, while pentoxifylline, a molecule that has been used for many years in the treatment of vascular diseases, possesses vasodilatory, platelet aggregation inhibiting, and anti-inflammatory properties [9,10,11].

The aim of our study was to evaluate the effects of a combined treatment with pentoxifylline and alpha-tocopherol on endometrial ultrasound parameters, pregnancy and live birth rates in patients with uterine and endometrial vascularization and/or proliferation disorders.

## 2. Materials and Methods

### 2.1. Patient Selection

This single-center retrospective study was conducted at the ART Center of the University of Liège, Citadelle Hospital, Belgium, between January 2020 and December 2023. This study was approved by the local ethics committee of the Citadelle Hospital under reference number 2146.

**Figure 1:**
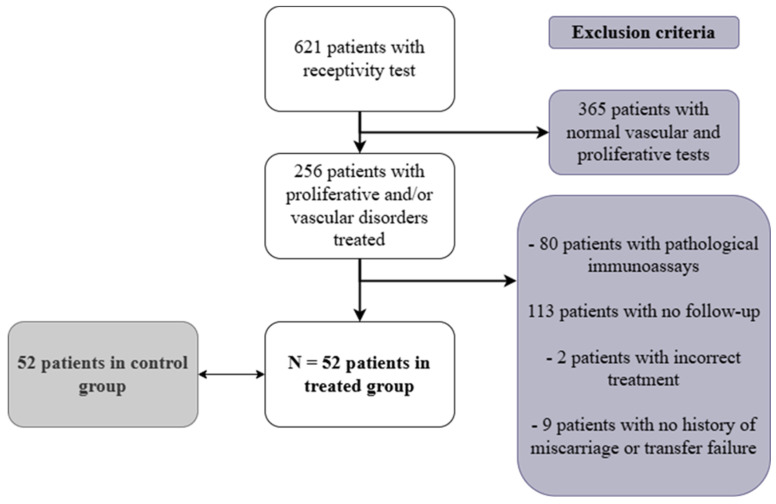
Study sampling: inclusion and exclusion criteria.

A total of 621 patients referred for RIF or RPL underwent ultrasound-based assessment of endometrial receptivity. Only those with abnormal ultrasound findings were included in the treated group. Among the 621 patients, 365 had a normal vascular and proliferative ultrasound assessment and were therefore not included in the treated group. As part of this evaluation, some patients also underwent endometrial immune profiling (MatriceLab Innove, Créteil, France). Patients with abnormal immune profiling were excluded from the study, as it could confound the evaluation of treatment effects. Additional exclusion criteria included failure to follow up (Figure 1).

A total of 52 patients who met the inclusion criteria were analyzed, and these were compared to a control group. The control group was selected from all fresh embryo transfers conducted between 2016 and 2023, at the ART Center of the University of Liège, Citadelle Hospital, Belgium. These control patients had not undergone prior receptivity testing and were matched with the treated group based on age, number of embryos previously transferred, and number of prior miscarriages at the time of transfer (Figure 1).

### 2.2. Endometrial Receptivity Testing and Ultrasound Measurements

All ultrasound measurements were performed using a Voluson S10 (GE Medical System, Machelen, Belgium) with a multi-frequency transvaginal probe. Three-dimensional power Doppler data were acquired following the methodology previously described by Lédée et al. [12], ensuring standardized settings across all cases. Volumetric and vascular assessments were conducted using the Virtual Organ Computer-aided Analysis (VOCAL) program to define the volume of interest and delineate the myometrial–endometrial border.

Endometrial proliferation was evaluated during the luteal phase based on endometrial volume (EV), considered pathological when <2 cm^3^. Vascularization was assessed by measuring the vascularization flow index (VFI) within the endometrium and sub-endometrium (within 5 mm of the border) and the pulsatility index (PI) of the uterine arteries. A PI greater than 2.8 in one or both uterine arteries (PI > 2.8) was considered abnormal, as was a VFI below 0.25 (VFI < 0.25). The ascending branch of each uterine artery was imaged in the vaginal fornices, and a pulsed Doppler gate was used to record flow velocity waveforms, with blood flow impedance expressed as the mean PI of both arteries.

Patients presenting at least one pathological criterion out of the three measured were considered to have a vascularization and/or endometrial proliferation disorder (*n* = 256).

### 2.3. Treatment Protocol

Patients with vascularization and/or endometrial proliferation disorders received a combination therapy consisting of pentoxifylline (400 mg twice daily) and alpha-tocopherol (500 IU twice daily).

**Figure 2:**
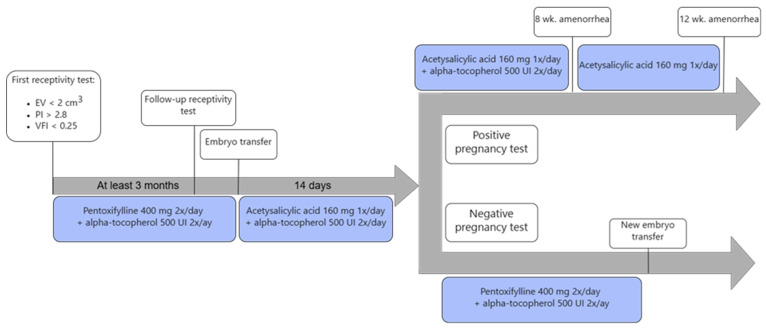
Study timeline. EV = Endometrial Volume; PI = pulsatility of the uterine arteries; VFI = vascularization flow index.

A follow-up appointment was scheduled at least three months later, during which a repeat endometrial receptivity test was performed, and ultrasound measurements were collected. Patients continued treatment until embryo transfer following this reassessment. As the safety of pentoxifylline during pregnancy has not been established, it was discontinued on the day of embryo transfer and replaced with acetylsalicylic acid (ASA). Alpha-tocopherol was continued until 8 weeks of amenorrhea in the case of a confirmed implantation and ASA until 12 weeks of amenorrhea. If implantation was not achieved, patients resumed the combined treatment with pentoxifylline (400 mg twice daily) and alpha-tocopherol (500 IU twice daily) until the next transfer cycle (Figure 2).

### 2.4. Outcome Measures

For all patients, the outcomes of the first embryo transfer after a minimum of three months of treatment were assessed, along with the outcomes of subsequent embryo transfers performed within six months of the initial transfer.

### 2.5. Statistical Analysis

Data from 52 patients in the treated group and 52 patients in the control group were analyzed. The results presented in the form of means, standard deviations (SD), quartiles (median, Q1, Q3) and extremes (minimum, maximum) for the quantitative variables and in the form of frequency tables for the qualitative variables.

To compare measurements between the two receptivity tests, the McNemar test was used for qualitative variables and the unpaired Student’s *t*-test for quantitative variables. To compare the characteristics of the two groups and their pregnancy rates, since the two groups were matched, matched tests were used, considering that patients in the same pair formed a single element (matched Student’s *t*-test, Wilcoxon signed ranks and McNemar’s test). Logistic regression and Poisson regression were used to compare groups by adjusting for the three matching parameters.

The results are considered significant at the 5% uncertainty level (*p* < 0.05). Calculations were performed using SAS version 9.4.

## 3. Results

### 3.1. Study Population

As described in the Materials and Methods section, the treated patient group (*n* = 52) was compared to a corresponding control group (*n* = 52). Several patient characteristics were collected and analyzed in both groups, with detailed results presented in Table 1.

Patients in the treated group presented a significantly higher AMH level than the control group (*p* = 0.0017). The average number of miscarriages was also significantly higher in the treated group (*p* = 0.0008). However, when considering the categories “0 to 1 miscarriage” and “2 or more miscarriages” for matching the control population to the treated one, the two groups were homogeneous (*p* = 1.00). With regard to other characteristics, there was no significant difference between the two groups (Table 1).

### 3.2. Effect of Pentoxifylline and Alpha-Tocopherol on Endometrial Vascularization and Proliferation

As described in Table 2, receptivity tests by ultrasound were carried out before (Test 1) and at least 3 months after a treatment with pentoxifylline and alpha-tocopherol (Test 2).

With regard to the proliferative side, the mean endometrial thickness increased significantly in the second assessment by 0.78 mm (*p* = 0.019), associated with a significant enhance of mean endometrial volume of 0.32 cm^3^ (*p* = 0.0054). On the vascular side, VFI increased significantly by 0.49 (*p* = 0.041) between the two tests. The PI of the right uterine artery decreased significantly by 0.25 (*p* = 0.029) and the PI of the left uterine artery decreased without signification by 0.27 (*p* = 0.083).

After treatments, the number of patients presenting proliferative disorder was significantly decreased (*p* = 0.02), as well as the number of patients with vascular disorder (*p* = 0.0004), compared with the results before treatment (Table 2).

### 3.3. Outcome of the First Embryo Transfer

Of the 52 treated patients, 9 did not undergo embryo transfer within 6 months of their second follow-up and 9 patients were lost to follow-up. Consequently, 34 treated patients had available embryo transfer results. As described in materials and methods section, only complete pairs (treated patient and her matched control) were included for a proper comparison.

The results of the first embryo transfer, conducted after at least 3 months of treatment and following the control receptivity test, were analyzed.

In the treated group, the clinical pregnancy rate (CPR) was 38.2%, with a live birth rate (LBR) of 17.7%. In comparison, the control group showed a CPR of 23.5% and a LBR of 14.7% (Table 3). However, these differences were not statistically significant.

The results of the embryo transfers within 6 months following the first embryo transfer were analyzed for both the treated and control groups. In the treated one, 50% of patients achieved at least one clinical pregnancy, compared with 35.3% in the control group (*p* = 0.17). In terms of LBR, 32.3% was observed in the treated group, compared to 29.4% in the control group (*p* = 0.78) (Table 4).

Although not statistically significant, the treated group exhibited higher rates of biochemical pregnancy, clinical pregnancy, and live birth within the first transfer, or within 6 months, compared to the matched control group (Table 3 and Table 4).

## 4. Discussion

This study aimed to evaluate the effects of a combined treatment with pentoxifylline and alpha-tocopherol on endometrial receptivity parameters and reproductive outcomes in women with suspected endometrial vascularization and/or proliferation disorders. These ultrasound-detected abnormalities, assessed during the implantation window, represent one aspect of a broader assessment of endometrial receptivity, which may include histological, immunological, ultrasound-based, microbiota or genome-derived parameters. This evaluation could be intended for patients with RIF and recurrent miscarriages. According to the ESHRE consensus, RIF is defined by the cumulative chance of implantation for a given patient or couple at a given time. If this chance is greater than 60%, RIF is considered after at least two transferred embryos fail to result in pregnancy [13]. RPL is defined as the loss of 2 or more pregnancies [14].

Ultrasound assessment of endometrial receptivity could be easily used in clinical practice due to its non-invasive and cost-effective nature, allowing the evaluation of endometrial vascularization and/or proliferation disorders. However, there is currently no consensus on the optimal treatment strategy for women presenting with such findings. Several options have been investigated, including estrogen supplementation, ASA, vitamins and, more recently, platelet-rich plasma (PRP), but their efficacy has yet to be proven [9].

Among the therapeutic options explored, pentoxifylline, a methylxanthine derivative, has long been used in the treatment of vascular diseases such as intermittent claudication for its vasodilatory, platelet aggregation inhibiting and anti-inflammatory properties. Alpha-tocopherol, for its part, has well-known antioxidant properties, by eliminating the free radicals generated during oxidative stress [9,10,11]. The combination of pentoxifylline and alpha-tocopherol was initially studied in the treatment of radiation-induced uterine damage in patients who had undergone radiotherapy, usually in childhood, for certain abdomino-pelvic cancers. These patients developed radiation-induced fibrosis, leading in particular to endometrial atrophy and arterial fibrosis. After treatment with alpha-tocopherol and pentoxifylline, a study showed a significant increase in endometrial thickness and an improvement in uterine vascularization (reduction in uterine artery PI) in these patients [9,10].

Moreover, previous studies have suggested that the combination of pentoxifylline and alpha-tocopherol may improve endometrial parameters in women with thin endometrium during ART, although these studies were limited by small sample sizes and heterogeneous protocols. In the study of Lédée et al., published in 2002, a significant increase in endometrial thickness was observed in patients who repeatedly presented with a thin endometrium (less than 6 mm) after the combined treatment [11]. In this study, the dose of pentoxifylline was 400 mg per day, and that of alpha-tocopherol was 500 IU per day. A study conducted in England in 2009 corroborated these results, using twice the dose in patients undergoing ART with a thin endometrium. Tolerance to the treatment was excellent despite the higher doses, with no patients reporting any side effects [15]. Theses doses were equivalent to those used in our center and none of the treated patient discontinued the treatment due to poor tolerance or the presence of side effects. These initial studies, although encouraging, all involved very small populations between 6 and 20 patients.

Nevertheless, in these studies, endometrial measurement was performed in two dimensions. Other teams have explored three-dimensional evaluation in order to assess the overall endometrial volume rather than just its thickness, which can be highly subjective depending on the measurement location. However, there is currently no consensus on the endometrial volume threshold that is considered to be pathological and responsible for implantation failure. Reported thresholds vary, ranging from 2 cm^3^ to 3.2 cm^3^ [5,16,17]. In our center, a pathological endometrial volume was defined as being less than 2 cm^3^, in accordance with the French study by Krief et al. [18].

In this more recent French retrospective study, involving 143 patients, a significant increase in endometrial volume following the combined treatment has been described, thereby reinforcing the relevance of three-dimensional assessment in evaluating therapeutic efficacy. In patients with an endometrial volume of less than 2 cm^3^ before treatment, they demonstrate an increase in endometrial volume of 0.47 cm^3^ after treatment with pentoxifylline 400 mg twice daily and alpha-tocopherol 500 IU twice daily for at least three months [18].

Our findings are consistent with and expand upon these earlier results. In our cohort, composed of patients referred for RIF or RPL, ultrasound-based receptivity assessments performed before and after at least three months of the combined treatment (pentoxifylline 400 mg twice daily and alpha-tocopherol 500 IU twice daily) revealed significant improvements in both proliferative and vascular parameters. Specifically, endometrial thickness significantly increased by 0.78 mm, and endometrial volume by 0.32 cm^3^. Vascularization also improved, with a significant increase in sub-endometrial VFI, and a reduction in the PI of uterine arteries, which was significant for the right one. A threshold of 0.25 was defined for abnormal sub-endometrial VFI, consistent with previous studies [7].

The primary objective of ART is to assist patients in conceiving. Therefore, the impact of this treatment on CPR and LBR was evaluated. Establishing a control population enabled the comparison of pregnancy rates between the treated patient group and those who had not undergone a receptivity test and did not receive treatment (control group). To ensure appropriate comparisons between the two groups, the populations were matched based on age, number of embryos previously transferred, and number of prior miscarriages. These criteria were applied to ensure one-to-one matching between treated and control patients. Age categories were defined to compare patients with similar implantation chances, considering that pregnancy rates per cycle decrease with age [19]. The control population had similar characteristics as the treated population in terms of smoking status, BMI, primary or secondary infertility and duration of infertility, partner sperm result, number of IVF/ICSI prior to the study and use of oocyte donation. The AMH of the control group was lower than that of the treated population but remained above 1.2 ng/mL, the threshold below which patients are considered poor responders in IVF cycles with ovarian stimulation according to the Poseidon criteria [20]. Consequently, even if the AMH was significantly different between the two groups, there was no clinical repercussions as the level was considered normal.

The benefits of the treatment on IVF results were already noticeable at the time of the first transfer carried out after treatment with alpha-tocopherol and pentoxifylline, although not significant, since treated patients had a CPR of 38.2% compared to 23.5% in untreated control group. In the 6 months following the first transfer, CPR rose to 50% for treated patients and 35.3% for the control patients, with an average of one transfer per patient. The analysis of LBR, six months after the control receptivity test, showed 32.3% in the treated group compared to 29.4% in the control group. These results are encouraging and suggest a potential benefit of the treatment in improving ART outcomes.

To our knowledge, this is the first study to specifically evaluate the impact of combined pentoxifylline and alpha-tocopherol treatment on pregnancy outcomes, highlighting the potential clinical relevance of these findings and the need for further prospective research. Nevertheless, this study has several limitations that should be acknowledged. First, its retrospective and single-center design may limit the generalizability of the findings. Second, the sample size was relatively small, particularly when analyzing pregnancy and live birth outcomes, which may have reduced the statistical power to detect significant differences. Third, although efforts were made to match the control group based on key clinical parameters, residual confounding factors cannot be entirely excluded. Finally, the exclusion of patients with abnormal immune profiling, while necessary to isolate the effect of the treatment, may have introduced a selection bias.

## 5. Conclusions

The management of patients experiencing RIF or RPL remains a significant clinical challenge. Although embryo quality is undeniably a key determinant of implantation success, the role of the endometrium should not be overlooked. In this context, receptivity testing represents a valuable component of the diagnostic and therapeutic strategy for patients facing repeated implantation failure or pregnancy loss. Despite growing interest in the endometrial contribution to implantation, there is still no consensus regarding the optimal treatment for patients presenting with vascular or proliferative endometrial abnormalities. It is essential to remember that the ultimate objective of ART is not only to achieve pregnancy, but to ensure a live birth.

In our study, combined treatment with alpha-tocopherol and pentoxifylline significantly improved endometrial vascularization and proliferation. Although the observed increases in pregnancy and live birth rates among treated patients did not reach statistical significance, the trend was favorable.

These encouraging results suggest a potential benefit of this therapeutic approach. However, confirmation through larger-scale studies is necessary to enhance statistical power and validate its impact on clinical outcomes, particularly pregnancy and live birth rates.

## Figures and Tables

**Table 1:** Characteristics of treated and control groups.

		Control Group	Treated Group		
Variable	N	Mean ± SD /Median [Q1–Q3] /Number (%)	Mean ± SD /Median [Q1–Q3] /Number (%)	*p*-Value	95% CI
Age (years)	52	35.06 ± 4.56	34.46 ± 4.70	0.22	[−1.92; 3.12]
Age (years) (categories) *	52			1.00	
<36		32 (61.5)	32 (61.5)		
36–39		8 (15.4)	8 (15.4)		
40–42		12 (23.1)	12 (23.1)		
>42		0 (0.0)	0 (0.0)		
Tobacco	48			0.13	[−0.029; 0.279]
No		42 (87.5)	36 (75.0)		
Yes		6 (12.5)	12 (25.0)		
BMI (kg/m^2^)	46	25.60 ± 4.93	24.48 ± 4.33	0.22	[−1.56; 3.80]
AMH (ng/mL)	45	1.31 [0.81–1.80]	1.77 [1.00–3.63]	0.0017	[−0.41; 1.33]
1st or 2nd infertility	52			0.36	[−0.109; 0.263]
Primary		20 (38.5)	24 (46.2)		
Secondary		28 (53.9)	27 (51.9)		
Couple of women		4 (7.7)	1 (1.9)		
Spermogram	46			0.99	
Teratozoospermia		12 (26.1)	12 (26.1)		
Cryptozoospermia		1 (2.2)	2 (4.4)		
Azoospermia		3 (6.5)	1 (2.2)		
Oligozoospermia		1 (2.2)	3 (6.5)		
Oligoasthenozoospermia		0 (0.0)	4 (8.7)		
Oligoteratozoospermia		7 (15.2)	5 (10.9)		
OAT		5 (10.9)	6 (13.0)		
Donor		9 (19.6)	6 (13.0)		
Normal		8 (17.4)	7 (15.2)		
Number of previous IVF/ICSI	51	2.41 ± 1.00	2.14 ± 1.64	0.22	[−1.01; 0.47]
Number of embryos previously transferred	52	3.94 ± 3.15	4.46 ± 3.48	0.18	[−2.32; 1.28]
Number of embryos previously transferred (categories) *	52			1.00	
0		9 (17.3)	9 (17.3)		
1–4		19 (36.5)	19 (36.5)		
5 and over		24 (46.2)	24 (46.2)		
Number of previous miscarriages	52	0.71 ± 1.00	1.15 ± 1.60	0.0008	[−1.17; 0.29]
Number of previous miscarriages (categories) *	52			1.00	
0–1		36 (69.2)	36 (69.2)		
2 and more		16 (30.8)	16 (30.8)		
Egg donation	52			0.16	[−0.0135; 0.0915]
Yes		0 (0.0)	2 (3.9)		

* Variables chosen for matching the treated population and the control population. N = number of complete pairs (treated patient and her control) for the studied parameters.

**Table 2:** Comparison between the two receptivity tests for the treated group (*n* = 52).

Variable	N	Test 1 Mean ± SD /Number (%)	Test 2 Mean ± SD /Number (%)	*p*-Value	95% CI/95% CI of the Difference
Exam performed under:	52			0.052	[0.005; 0.263]
Artificial cycle		49 (94.2)	42 (80.8)		
Natural cycle		3 (5.8)	10 (19.2)		
Endometrial thickness (mm)	52	6.63 ± 2.15	7.41 ± 2.38	0.019	[0.13; 1.43]
Endometrial volume (cm^3^)	51	1.57 ± 0.95	1.89 ± 1.12	0.0054	[0.10; 0.54]
Endometrial volume > 2 cm^3^	51	10 (19.6)	17 (33.3)	0.035	[−0.006; 0.280]
Endometrial volume > 2.5 cm^3^	51	9 (17.6)	11 (21.6)	0.48	[−0.114; 0.194]
Endometrial volume > 3.2 cm^3^	51	3 (5.9)	7 (13.7)	0.10	[−0.036; 0.192]
Sub-endometrial VFI	49	1.00 ± 1.52	1.50 ± 1.74	0.041	[0.02; 0.96]
Sub-endometrial VFI > 0.25	49	33 (67.3)	44 (89.8)	0.0076	[0.086; 0.364]
IP right uterine artery	51	2.32 ± 0.70	2.07 ± 0.54	0.029	[−0.48; −0.02]
IP left uterine artery	52	2.53 ± 0.99	2.27 ± 0.79	0.083	[−0.57; 0.03]
IP of the uterine arteries	51			0.083	[−0.005; 0.241]
0 PI > 1.8		3 (5.9)	9 (17.7)		
1–2 IP > 1.8		48 (94.1)	42 (82.3)		
Proliferation disorder present	49	40 (81.6)	31 (63.3)	0.020	[−0.326; −0.040]
Vascularization disorder present	49	28 (57.1)	11 (22.4)	0.0004	[−0.495; −0.199]

N = Number of patients in the treated population for whom the parameter is available in both tests.

**Table 3:** Outcome of the first embryo transfer in the treated and control groups.

Variable	N	Control Group Number (%)	Treated Group Number (%)	*p*-Value	95% CI of the Difference
hCG	34				
Positive		9 (26.5)	13 (38.2)	0.28	[−0.104; 0.338]
CPR	34				
Yes		8 (23.5)	13 (38.2)	0.17	[−0.069; 0.363]
LBR	34				
Yes		5 (14.7)	6 (17.7)	0.72	[−0.145; 0.205]

N = number of patients for whom information is available; CPR: clinical pregnancy rate, LBR: live birth rate.

**Table 4:** Cumulative outcomes per patient within 6 months of the first embryo transfer in the control and treated groups.

Variable	N	Control Group Number (%)	Treated Group Number (%)	*p*-Value	95% CI of the Difference
Number of transfers carried out over 6 months	43				
0		0 (0.0)	9 (20.9)		
1		21 (40.4)	25 (58.1)		
2		21 (40.4)	6 (14.0)		
3		7 (13.5)	3 (7.0)		
4		2 (3.8)			
5		1 (1.9)			
hCG	34				
Positive		12 (35.3)	18 (52.9)	0.12	[−0.056; 0.408]
Clinical pregnancy	34				
Yes		12 (35.3)	17 (50.0)	0.18	[−0.085; 0.379]
Live birth	34				
Yes		10 (29.4)	11 (32.3)	0.77	[−0.190; 0.248]

N = number for whom information is available.

## Data Availability

The original contributions presented in this study are included in the article. Further inquiries can be directed to the corresponding author.
